# How Do B-Learning and Learning Patterns Influence Learning Outcomes?

**DOI:** 10.3389/fpsyg.2017.00745

**Published:** 2017-05-16

**Authors:** María Consuelo Sáiz Manzanares, Raúl Marticorena Sánchez, César Ignacio García Osorio, José F. Díez-Pastor

**Affiliations:** ^1^Department of Health Sciences, University of BurgosBurgos, Spain; ^2^Department of Civil Engineering, University of BurgosBurgos, Spain

**Keywords:** learning analytics, learning management systems, blended learning, supplemental blend, replacement blend, successful learning, self-regulated learning, learning outcomes

## Abstract

Learning Management System (LMS) platforms provide a wealth of information on the learning patterns of students. Learning Analytics (LA) techniques permit the analysis of the logs or records of the activities of both students and teachers on the on-line platform. The learning patterns differ depending on the type of Blended Learning (B-Learning). In this study, we analyse: (1) whether significant differences exist between the learning outcomes of students and their learning patterns on the platform, depending on the type of B-Learning [Replacement blend (RB) vs. Supplemental blend (SB)]; (2) whether a relation exists between the metacognitive and the motivational strategies (MS) of students, their learning outcomes and their learning patterns on the platform. The 87,065 log records of 129 students (69 in RB and 60 in SB) in the Moodle 3.1 platform were analyzed. The results revealed different learning patterns between students depending on the type of B-Learning (RB vs. SB). We have found that the degree of blend, RB vs. SB, seems to condition student behavior on the platform. Learning patterns in RB environments can predict student learning outcomes. Additionally, in RB environments there is a relationship between the learning patterns and the metacognitive and (MS) of the students.

## Highlights

– Good teaching designs in Learning Management Systems (LMS) encourage the development of process-oriented feedback.
– Learning Analytics (LA) allow a prediction of the learning patterns of at-risk students.
– Different learning patterns in B-Learning environments, Replacement blend (RB) vs. Supplemental blend (SB).
– Successful learning in the LMS depends on the design of teaching.
– Metacognitive strategies are related with the type of learning pattern in LMS.
– Learning patterns differ in accordance with the type of task.

## Introduction

### Learning management systems and improvements to the learning process

Over recent years, Learning Management Systems (LMS) have been very effectively used in teaching-learning processes, especially in Higher Education. LMS have been related to improvements in learning outcomes and in information acquisition (Cerezo et al., 2016). These systems have the objective of introducing improvements in the learning process, through the use of new technologies (de Raadt et al., 2009; Xinogalo, 2015), because:

1. They strengthen the development of Self-Regulated Learning (SRL), which increases student motivation. Likewise, the use of LMS reduces abandonment and leads to more successful learning outcomes (Schraw et al., 2007).
2. They allow the teacher to provide the student with more immediate feedback. They also make it possible to register both the teacher's feedback actions and the student's queries about these actions (Sáiz and Marticorena, 2016).
3. They facilitate collaborative and constructive learning (Zacharis, 2015; Yücel and Usluel, 2016).

Also in the context of SRL, LMS provide students with the possibility of developing scaffolding that strengthens planning, monitoring, control and reflection on the object of learning. Likewise, LMS increase understanding and the construction of learning (Azevedo, 2005). Recent investigations (Winne, 2014; Höök and Eckerdal, 2015) have highlighted those individuals who learn with scaffolded tasks of growing difficulty increase autonomy in problem-solving processes. In summary, the stepped structure of learning permits the learner to sequence both goals and the steps needed for task-related problem-solving. LMS not only facilitates the stepped structure of learning, but it also increases motivation toward the object of learning and SRL (Segedy and Biswas, 2015).

An essential aspect in the whole process is the feedback that the teacher provides through the platform. On this point, it is necessary to differentiate two types of feedback: (1) process-oriented feedback, includes the motivational, the cognitive and the metacognitive characteristics of students that are taken into account by the teacher for the design of the feedback; and, (2) grade-oriented feedback, which refers to information on the execution of the learning task or process (incorrect, correct or excellent), but does not descend to the aspects involved in process-oriented feedback (Hattie and Timperley, 2007; Harks et al., 2014). The first type of feedback is more effective, as it facilitates the construction and scaffolding of knowledge (Hattie, 2013; Mentzer et al., 2015).

Well-designed LMS mean that the development of process-oriented feedback is more structured for students, since they can consult the orientations of the teacher, both in real time and afterwards, at any time in the learning process (Sáiz and Marticorena, 2016).

With regard to collaborative learning, this investigation highlights that LMS increase and improve problem-solving routines and increase metacognitive strategies for efficient problem solving (Bernard and Bachu, 2015; Malmberg et al., 2015; Järvelä et al., 2016; Sáiz and Marticorena, 2016). Although, according to Bernard and Bachu (2015), the teacher must start by analyzing students' prior knowledge and clearly formulating the tasks they have to carry out. The teacher also has to provide student with help guides which clearly reflect the objectives, planning of executions and deliveries. These tasks must have an increasing degree of difficulty in order to avoid students' dropouts. Likewise, the teacher should provide accurate feedback of the tasks. All this will increase the motivation of the students.

Nevertheless, the mere use of LMS will not guarantee better results in the teaching-learning process. On the one hand, any such use is conditional upon the design that the teacher makes of the learning activities, as well as the type of feedback that the teacher provides on the evidence of learning. On the other hand, the teacher has to perform an analysis of the patterns of learning behavior of the students. Recent studies have indicated that for a satisfactory development of the teaching-learning process in LMS, training in their use is necessary both for teachers and for students (Yamada and Hirakawa, 2015), given that the mere use of the platforms cannot in itself guarantee the effectiveness of the learning process. Park and Il-Hyun (2016) found significant differences, studying variables, such as the characteristics of teachers, of students, and the structuring and the design of subject modules.

Another relevant aspect in this learning process in LMS is the use of tools for analyzing the log records registered by the platform for the early detection of students at-risk of obtaining poor academic results. Recent studies (Zacharis, 2015; Strang, 2017) have analyzed the relation between the use of the LMS and the behavioral patterns of learning among students. Both successful and at-risk conducts may be detected with regression analysis techniques. Those conducts explain up to 52% of the variance in the learning outcome. The studies are validated through the use of data-mining techniques supported by the use of a well-known tool: Waikato Environment for Knowledge Analysis; better known by its WEKA acronym (Frank et al., 2016). According to, some authors (Zacharis, 2015; Cerezo et al., 2016), the learning behavior that is considered key in the analysis of behavioral patterns of learning are:

1. General effort.
2. Time spent performing the tasks.
3. Working time on theoretical contents.
4. Results in the self-evaluation tests.
5. Time spent in the discussion groups on the forums.
6. Quality of the discussions in the forums (type of message and its length).
7. Time spent analyzing the feedback provided by the teacher.
8. Number and type of messages sent.
9. Frequency of use of the LMS.
10. Accessing the forums to read messages.
11. Contributing to the creation of content.
12. Number of files accessed.
13. Effort required in quizzes.
14. Handing in assignments on time.

### Logs, learning analytics, and educational data mining

In LMS, the interactions of all user roles (student, teacher and administrator) are recorded in log files. These logs may be analyzed and the use of data-mining techniques allows patterns to be discovered or new information to be extracted from these large datasets. We refer to Learning Analytics (LA) or Educational Data Mining (EDM) when these techniques are employed with data relating to learning. These concepts are closely related between each other, although the first centers more on understanding the learning process, and the second of the two models allows us to analyse these data (Baker and Inventado, 2014).

Moodle is one of the most frequently used within the LMS (Dougiamas and Taylor, 2003). A Learning Management System (LMS) with a modular structure, it allows different resources to be used for different student (individual and group) and teacher profiles. It also means that different learning activities and actions (discussion forums, questionnaires, workshops, wikis, access to repositories) may take place and innovative teaching methods may be used, such as Project-Based Learning (PBL). The interactive behaviors that can be analyzed in this type of LMS, are as follows (Yücel and Usluel, 2016):

1. Student-student interaction.
2. Student-teacher interaction.
3. Student-content interaction.
4. Student-system interaction.
5. Teacher-student interaction.

Yücel and Usluel (2016) pointed out that it is important to consider the type, the quantity and the quality of the interaction. The use of each of these interactive conducts is reflected in the file of records or logs. Moodle permits the extraction of these files, where all of the different events and interactions between the members of the community of learning are stored, in order to facilitate an analysis that will provide a lot of information on the learning behavior of the users.

The information that may be obtained from the Moodle records is very extensive, which is why EDM has to be used to extract what is needed in each case (Iglesias-Pradas et al., 2015). So, there are techniques and models in EDM that will provide records of access: patterns of learning behavior among students and the interactions between them, as well as between the teacher and the students. Likewise, they provide methods for the extraction of information in real time. All of these, records support the processes of educational evaluation by the teacher. EDM can be applied to different roles (Romero and Ventura, 2007; Romero et al., 2013):

1. Oriented toward students: this approach is focused on learning tasks and the objective is the improvement of student learning.
2. Oriented toward educators: the objective is to provide feedback for instruction, to evaluate the course structure and its contents, to analyse elements that have been effective in the learning processes, to classify the type of students and to perceive the needs for guidance and monitoring of learning, the most common patterns in their learning, and the frequency of errors with a view to finding more effective activities.
3. Oriented toward responsible academics and administration: the objective is to provide information to the institution that will help it to improve its learning platforms.
4. At present, importance is given to the use of LA in LMS (Chatti et al., 2012; Agudo-Peregrina et al., 2014). As mentioned earlier, LA are a concept related with EDM, but their focus is more on understanding the learning process (Roberts et al., 2016). It can be used to investigate the responses to such questions as:

1. What data can be used? (*What*) As previously mentioned, an astonishing amount of information is recorded in the LMS, for which purpose the detection of patterns of data analysis is required (LA).
2. For whom is the information provided? (*Who*) It is important to distinguish the group to which the analysis is directed (students, teachers, tutors/mentors, educational administrators, etc.). For example, if we center on the students, the institution could be interested in knowing how to improve its systems to construct more effective areas of learning. Likewise, the teachers might wish to make their teaching practice more effective and to offer the support that their students need. The institutions would therefore be interested in detecting at-risk students and increasing the success of their students, for the purpose of taking administrative decisions on performance. In summary, educational managers increasingly propose the implementation of tools that offer data analysis for non-experts through the application of EDM. These techniques offer goal-oriented feedback that allows the user of the platform to reflect on the data for decision-taking.
3. Why is the information provided? (*Why*) There are different objectives in accordance with the role of the user. LA include: monitoring and analysis, in other words, the follow up of students so as to generate reports for the teacher and/or for the institution. The evaluation of student learning processes by the teacher will improve the learning environment. The prediction of the knowledge of the student and of the results of learning will permit the detection of at-risk students and give students the specific assistance that they need for successful learning. Likewise, tutoring and mentoring will facilitate process-oriented feedback.
4. How is the information provided? (*How*) The methods for the detection of the hidden learning patterns in LMS are:

1. Statistical methods: LMS permit the extraction of reports based on the interaction of the teacher and the students on the platform that analyses time online, total number of visits, number of visits per page, distribution of visits over time, frequency of replies, etc. The statistical analyses that LMS provide are mean (*M*) and standard deviations (*SD*).
2. Visual information: set out reports of the data distributions that are easy to interpret by users.
3. Data mining: the methods are fitted into categories of supervised learning (classification or regression), non-supervised (clustering) and data association rules.
4. *Classification* (supervised learning): processes of finding a function or a model that distinguishes the data classes. The classes associated with each object are known during the training process. The objective is subsequently to predict the classes of objects with an unknown class label. It includes decision trees, neuronal networks, Bayesian classification and support vector machines together with the k-nearest neighbor classification.
5. *Clustering* in contrast with classification, the membership of a class in each training object or instruction is not well-known. The data are organized into groups from the criterion of inclusion in similar clusters vs. different clusters. The criterion is how the data are situated in the multi-dimensional space defined by the values of attributes and is based on a function of distance. The clustering methods are usually classified by partitioning methods and hierarchical methods. In the first, each participation represents a cluster. The technique begins with an arbitrary partition and then objects are changed from one group to another. Popular heuristic methods are used, such as the k-means algorithm (each cluster is represented from the mean value of the objects in the cluster), and the k-medoids algorithm (each cluster is represented by one of the objects of the cluster). Hierarchical methods create a hierarchy of groups in the form of a tree. The hierarchical clustering algorithms can be of two types: agglomerative and divisive: in the agglomerative algorithms, the hierarchy of groups is organized in an ascendant way (bottom up or by fusion merging): Initially each group is formed by a single observation, then these groups merge to form larger ones and so on, until all observations are in a single group. In the divisive algorithms, the clustering process is organized in a descendent manner (top down or splitting): initially all the observations belong to the same cluster and subsequently they are split in a recursive way.

Educational Data Mining (EDM) is multidisciplinary, in which techniques of algorithm construction, artificial neural networks, instance-based learning, Bayesian learning, programming techniques and statistical techniques all converge and different analytical procedures may also be used. These procedures may be grouped into clustering techniques, outlier detection techniques, association rule mining, sequential pattern mining and text mining (Romero and Ventura, 2007).

In summary, the use of the different techniques in EDM depends on the objectives of the task analysis. Nevertheless, investigators need to find the pedagogical objectives that are needed in the prediction, as well as the recommendations that are pertinent in each case. The challenge of the data analysis techniques centers on the analysis of tasks that allow feedback to be given to the teachers and solutions to be able to intervene in the learning process in an early and effective manner.

Another aspect that has to be considered is that the behavioral patterns on the platform depend on the type of B-Learning (Margulieux et al., 2016). For example, in Replacement blend (RB) (feedback given on the learning production on the platform), participation in the discussion forums is essential, while this aspect is not as important in Supplemental blend (SB) (feedback given on the Face-to-Face (F2F) productions), because the interaction may be done F2F (Cerezo et al., 2016). Another variable is that not all the students have the same learning process in the LMS. Likewise, another relevant indicator is that the teaching on the LMS can be designed in either a traditional or an innovative way (team-based projects, online discussion forums and online quizzes; Park and Il-Hyun, 2016).

In addition, it is necessary to carry out an evaluation of user satisfaction (students and teachers), employing the LMS (Hornbæk, 2006). The e-evaluation models suggest that there are different variables that have an influence: personal factors, behaviors that the students develop, and the environment in which the learning takes place (Harrati et al., 2016). Likewise, different learning patterns have been found, depending on the type of evaluation carried out by the teacher, which is directly related with the learning outcomes.

The use of the methodologies described above allows patterns and new information to be detected on the basis of data sets, such as the log files for example. In this study, we are particularly interested in responding to the following research questions:

RQ1: Will the learning patterns of students on the platform differ depending on the structure of the training program (RB vs. SB)?

RQ2: Will a relation be found between the learning patterns of students on the platform and the learning outcomes?

RQ3: Will a relation exist between the learning outcomes, the patterns of learning of the students on the platform, the metacognitive and the (MS) of students?

RQ4: Will the learning behaviors of the students on the platform offer different learning patterns?

## Materials and methods

### Participants

We worked with a sample of 129 students, 69 students on the first-year Computer Science Degree (CSD), who were following intermediary subjects on the degree course and 60 students from the branch of Health Sciences, 41 students from the Occupational Therapy Degree (OTD) and 19 on the Nursing Degree (ND) who were studying at intermediary levels on the degree course. In Table 1, the gender and the mean age of everybody in the groups may be seen.

**Table 1:** **Descriptive statistics of the variables assigned age and gender**.

**Degree**		**Men**			**Women**		
	**N**	***n***	***M_*age*_***	***SD_*age*_***	***n***	***M_*age*_***	***SD_*age*_***
Computer Science Degree (CSD)	69	60	20.34	1.42	9	21.55	2.5
Nursing Degree (ND)	19	3	26.66	8.19	16	26.26	8.19
Occupational Therapy Degree (OTD)	41	7	23.28	2.92	34	22.58	1.55

*N, total number of participants; n, number of participants by variable (sex and degree); M_age_, Mean age; SD_age_, Standard deviation by age*.

### Instruments

The following techniques and tools were used in this experimental project.

1. The Moodle platform (version 3.1). It was used to analyse records on: 1. Access to complementary information; 2. Access to theory; 3. Access to practice; 4. Access to self-evaluation; 5. Access to feedback given by the teacher; 6. Participation in the forum; 7. Mean access rate per day.
2. The Scale of learning strategies (ACRAr) by Román and Poggioli (2013). This scale is a highly tested instrument in investigations on learning strategies in Spanish-speaking populations (Camarero-Suárez et al., 2000; Carbonero et al., 2013) and identifies 32 strategies at different times of processing the information. The list of the scales with the indexes of validity is presented in Table 2. In this study, only the metacognitive scales were used and the strategies of motivation within the scale of support for information processing.
3. In this study, the indicators of validity in the scales used in the sample were: Metacognition α = 0.92 and Information processing support α = 0.91.
4. Learning outcomes among the students with OTD, ND, and CSD. The results of learning theoretical aspects (exam test that in all groups consisted of a multiple choice test-type exam with one true answer) and practical aspects. However, in the two first groups, when working for (PBL) both preparation and defense were considered.

**Table 2:** **Strategies in each one of the ACRAr scales (Román and Poggioli, **2013**) and of the different validity coefficients**.

**ACRAr Scales**	**Type of strategies**	**Number of strategies**	**Inter-judge validity**	**Construct validity**	**Content validity**
Acquisition of information	Repetition and re-reading	6	α = 0.78	*r* = 0.75	*r* = 0.85
Encoding information	Mnemonics, organization and preparation	12	α = 0.92	*r* = 0. 86	*r* = 0. 87
Recovery of information	Search and generation of responses	4	α = 0.83	*r* = 0. 86	*r* = 0. 86
Metacognition	Self-knowledge, self-planning and regulation and self-evaluation	4	α = 0.90	*r* = 0. 88	*r* = 0. 88
Information processing support	Self-instructions, self-control, counter-distractions, social interventions, intrinsic and extrinsic motivation, and escapist motivations	6	α = 0.90	*r* = 0. 88	*r* = 0. 88

### Procedure

Before starting the study, students were passed information and invited to participate in the project, so that their participation was voluntary. In CSD, a pair-based working methodology was used. The subject module on the Moodle platform was structured into: mandatory working material (theory); complementary material; guided practical laboratory sessions; with follow up assignments; self-evaluation activities (questionnaires) and two mandatory practical activities. The teacher returned feedback both on the laboratory assignments and the completion of the practical work through the platform. Likewise, the students had to answer a pair of individual evaluation tests.

In the OTD and ND, the teaching was developed by using the project-based learning methodology (PBL). The Moodle assignment was structured into: Mandatory working material (theory), complementary material, practical activities (five) and the answers to the PBL and self-evaluation activities (questionnaires). Both the practices and the project were done in groups (3 or 5 students). The teacher provided F2F feedback. Likewise, the students had to answer an individual test-type exam.

In all the groups, the teaching methodology was based on self-regulation of learning following a guided structure of the learning process through successive approaches to the goal, facilitating self-evaluation activities and process-oriented feedback, through individualized follow-up of the work of each student.

In all cases, the subject modules had a duration of 14 weeks and the type of teaching was mixed (partly F2F and partly through the Moodle Platform). However, in the CSD Group, the teaching was structured around continuous use of the platform, including the F2F part, the interaction fundamentally taking place through assignments and process-oriented feedback online, and in the Group of Health Sciences, the F2F part was through in-person interaction. When the teaching for all the groups came to an end, they were given the Scale of metacognitive strategies and the ACRAr Scale of process support Strategies.

### Design, variables, and statistical analysis

These three elements of the study are defined as follows:

1. Designs: To respond to RQ1, a quasi-experimental design with no control group was used. And to respond to RQ2, RQ3 and RQ4, a descriptive-correlational design was used.
2. Variables: For the first design, the independent variable was the type of B-Learning (RB vs. SB) and the dependent variables were the patterns of learning behavior on the platform. In the second design, the variables were the patterns of learning behavior on the platform, the metacognitive strategies, the motivational strategies, and the learning outcomes.
3. Statistical analysis: (1) analysis of asymmetry and kurtosis. (2) Discriminant analysis. (3) Single-factor fixed-effect ANOVA (type of B-Learning), value of the effect (eta squared) and Bonferroni test. (4) Pearson correlations matrix. (5) Cluster analysis.

## Results

### Previous statistical analysis

Before starting the study, it was confirmed whether the sample of individuals followed a distribution within the parameters of normality. To do so, the values of asymmetry and kurtosis were found for the selected indicators: in asymmetry, the highest values|2.00| indicate extreme asymmetry and the lowest values indicate a normal distribution (Bandalos and Finney, 2001). With regard to kurtosis, values of between |8| and |20| suggest extreme kurtosis (Arias, 2008; Arias et al., 2013). In asymmetry, values were found within an interval of |0.03| to |1.74| and in kurtosis between |0.02| and |4.40|, which suggests that there is no serious deviation, from normality in the distributions (see Table 3).

**Table 3:** **Analysis of normality in the independent variables before the intervention**.

	**Group 1 (CSD)** ***n*** = 41								**Group 2 (ND)** ***n*** = 19								**Group 3 (OTD)** ***n*** = 69							
	***Min***	***Max***	***M***	***SD***	***AS***	***AE***	***KS***	***KE***	***Min***	***Max***	***M***	***SD***	***AS***	***AE***	***KS***	***KE***	***Min***	***Max***	***M***	***SD***	***AS***	***AE***	***KS***	***KE***
1. Access to complementary information.	0	10	3.76	2.39	0.48	0.37	0.27	0.72	0	22	8.37	6.42	0.85	0.52	0.02	1.01	0	37	9.84	6.97	1.74	0.29	4.24	0.57
2. Access to theory.	0	37	10.39	6.90	1.43	0.37	4.40	0.72	5	31	14.11	7.89	0.85	0.52	−26	1.01	1	69	34.71	14.78	0.26	0.29	0.07	0.57
3. Access to co-evaluation.	0	42	13.15	7.95	1.58	0.37	4.16	0.72	3	37	16.84	8.42	0.95	0.52	0.81	1.01	0	61	17.03	12.40	1.27	0.29	2.33	0.57
4. Access to feedback provided by the teacher.	0	114	23.56	25.58	1.64	0.37	2.93	0.72	0	25	8.05	6.66	0.86	0.52	0.67	1.01	1	71	160.41	170.49	1.56	0.29	2.17	0.57
5. Participation in the forum.	0	87	18.07	23.31	1.56	0.37	1.79	0.72	0	72	25.32	23.35	0.83	0.52	0.36	1.01	0	13	82.94	24.44	−1.03	0.29	2.36	0.57
6. Mean access rate per day.	0	14	5.22	2.91	1.25	0.37	2.21	0.72	0	9	3.89	2.56	0.40	0.52	0.60	1.01	0	54	11.84	14.36	1.48	0.29	1.54	0.57

*OTD, Occupational Therapy Degree; ND, Nursing Degree; CSD, Computer Science Degree; Min, minimum value; Max, maximum value; M, Mean; SD, Standard Deviation; AS, Asymmetry Statistic; AE, Asymmetry Error; KS, Kurtosis Statistic; KE, Kurtosis Error*.

In view of the results, a parametric statistic was used. The results of each research question are described below.

### Will the learning patterns of students on the platform differ depending on the structure of the training program?

In relation to the first research question (Will the learning patterns of students on the platform differ depending on the structure of the training program (RB vs. SB?), a total of 20,217 records were detected for the students from Health Sciences (OTD and ND), 13,847 in the case of OTD and 6,370 in the case of ND and 66,848 records were logged for CSD. These data are already indicative of different patterns of use of the platform by students from the three groups. Subsequently and to test whether the groups behaved in a different way in view of the learning behavior pattern on the platform, a discriminant analysis was performed. The results indicate that the behavior of the three groups differed in relation to all the indicators, except for the records of access to information on the theoretical contents of the subject modules that the students completed. As may be seen in Table 4, all of the Wilks' Lambdas are significant for all the indicators except for the records of access to theory. Likewise, the general *Lambda* (_14, 240_) = 15.29, *p* = 0.000 was significant with a high effect value $\eta_{\text{p}}^{2}$ = 0.47, which implies that the type of learning behavior pattern on the platform explains 47.1% of the variance among students.

**Table 4:** **Discriminant analysis between groups (OTD, ND, and CSD)**.

**Discriminant variables**	***Wilks' Lambda***	***F***	***Gl1***.	***gl2***	***p***
1. Access to complementary information.	0.82	14.20	2	126	0.000^**^
2. Access to theory.	0.97	1.83	2	126	0.165
3. Access to practice.	0.51	61.19	2	126	0.000^**^
4. Access to co-evaluation.	0.76	20.38	2	126	0.000^**^
5. Access to feedback provided by the teacher.	0.36	110.33	2	126	0.000^**^
6. Participation in the forum.	0.90	7.02	2	126	0.001^**^
7. Mean access rate per day.	0.55	51.77	2	126	0.000^**^

** *p < 0.01*.

**Figure 1:**
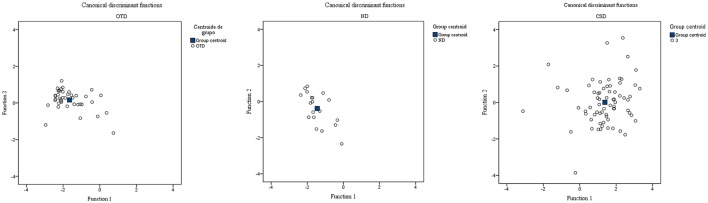
**Canonical discriminant functions of the patterns of learning behavior on the platform between the OTD, ND, and CSD groups**. OTD, Occupational Therapy Degree; ND, Nursing Degree; CSD, Computer Science Degree.

Subsequently, the canonical functions in each of the groups were found. The results show a different pattern in the learning behaviors, as may be confirmed in Figure 1. Greater dispersion of the individual students may be seen in the CSD, while student behavior in relation to the variables under analysis in the CSD and OTD is more homogeneous and the similarity of the centroids of the group is greater.

Next, with a view to studying whether significant differences existed between the three groups, a single factor, fixed-effects ANOVA was completed (type degree course). Significant differences were found in all the indicators except in the records of access relating to information on theory by the students (see Table 5). Subsequently, a Bonferroni test was carried out to study between which groups and in which indicators those differences were found. As may be appreciated from Table 6, the differences are found between students of CSD and ND and OTD in all the variables of behavior on the platform, except for the records of access to complementary information, in which a difference is appreciated between ND and CSD vs. OTD. It may therefore be concluded that the behavior of students of Health Sciences (OTD and ND) differs from the behavior of the CSD.

**Table 5:** **Single factor fixed-effects ANOVA (Type of group) and value of the effect**.

	**OTD *n* = 41**	**ND *n* = 19**	**CSD *n* = 69**			
	***M(SD)***	***M(SD)***	***M(SD)***	***F***	***p***	***η^2^***
1. Access to complementary information.	3.76 (2.39)	8.37 (6.42)	9.84 (6.40)	14.20	0.00^**^	0.18
2. Access to theory.	13.15 (7.95)	16.84 (8.42)	17.03 (12.40)	1.83	0.16	0.03
3. Access to practice.	10.39 (6.90)	14.11 (7.89)	34.71 (14.78)	61.19	0.00^**^	0.49
4. Access to co-evaluation.	23.56 (25.58)	8.05 (6.66)	160.41 (170.49)	20.38	0.00^**^	0.24
5. Access to feedback provided by the teacher.	18.07 (23.31)	25.32 (23.35)	82.94 (24.44)	110.33	0.00^**^	0.64
6. Participation in the forum.	5.22 (2.91)	3.88 (2.56)	11.84(14.36)	7.02	0.00^**^	0.10
7. Mean access rate per day.	1.05 (0.58)	1.296 (0.67)	3.21 (1.48)	51.77	0.00^**^	0.45

** *p < 0.01. OTD, Occupational Therapy Degree; ND, Nursing Degree; CSD, Computer Science Degree; M, Mean; SD, Standard deviation; η^2^ = eta squared (effect value)*.

**Table 6:** **Bonferroni test of differences of means between the OTD, ND and CSD**.

	**OTD vs. ND**		**OTD vs. CSD**		**ND vs. CSD**	
	***DM***	***p***	***DM***	***p***	***DM***	***p***
1. Access to complementary information.	−4.61	0.015	−6.08	0.000	–	–
2. Access to theory.	–	–	–	–	–	–
3. Access to practice.			−27.27	0.00^**^	−25.76	0.00^**^
4. Access to co-evaluation.	–	–	−136.85	0.00^**^	−152.35	0.00^**^
5. Access to feedback provided by the teacher.	–	–	−64.87	0.000^**^	−57.63	0.00^**^
6. Participation in the forum.	–	–	−6.62	0.00^**^	−7.95	0.02^*^
7. Mean access rate per day.	–	–	−2.16	0.00^**^	−1.91	0.00^**^

*OTD, Occupational Therapy Degree; ND, Nursing Degree; CSD, Computer Science Degree; DM, Difference of Means; p, Probability*.

* *p < 0.05*.

** *p < 0.01*.

### Interrelations between learning behaviors, metacognitive skills and motivation, and learning outcomes

Different patterns of learning having been detected on the platform, the results between the group of students of health sciences (OTD and ND) and the group of computer engineering students (CSD) were studied, in order to analyse the second research question (Will a relation be found between the learning patterns of students on the platform and the learning outcomes?) and the third research question (Will a relation exist between the learning outcomes, the patterns of learning of the students on the platform, the metacognitive and the (MS) of students?).

With regard to the group of students studying Health Sciences (OTD and ND), a KMO = 0.74 y χ^2^=225.85, *p* < 0.001 was found. As may be seen from Table 7, significant correlations were found between the learning outcomes in the different tests (*r* = 0.80, *p* < 0.01, *r* = 0.39, *p* < 0.01, *r* = 0.51, *p* < 0.01). Significant correlations were also found between the learning outcomes, except between SSM (Self-knowledge Metacognitive Skills) and LODPBL (Learning outcomes in the defense of PBL), and the SSM, Planning Metacognitive Skills (PMS), Evaluation Metacognitive Skills (EMS), and Motivational Strategies (MS). But no significant correlations were found between the patterns of learning, the learning outcomes in the different tests, the metacognitive skills and the motivational strategies.

**Table 7:** **Correlations matrix in the Health Sciences (OTD and ND) and the behaviors on the platform and the metacognitive skills and the motivational strategies**.

	**1**	**2**	**3**	**4**	**5**	**6**	**7**	**8**	**9**	**10**	**11**	**12**	**13**
LOPPBL	−												
LODPLBL	0.80^**^	−											
TELO	0.39^**^	0.50^**^	−										
SMS	0.22	0.26^*^	0.41^**^	−									
PMS	0.44^**^	0.59^**^	0.52^**^	0.39^**^	−								
EMS	0.45^**^	0.43^**^	0.51^**^	0.46^**^	0.69^**^	−							
MS	0.38^**^	0.47^**^	0.37^**^	0.18	0.37^**^	0.41^**^	−						
ACI	0.16	0.17	0.34^**^	0.10	0.19	0.19	0.27^*^	−					
AP	−0.02	−0.09	0.04	0.08	0.001	−0.12	0.19	0.46^**^	−				
AT	−0.12	−0.16	0.09	0.10	−0.01	−0.03	0.21	0.53^**^	0.67^**^	−			
ASA	0.20	0.16	−0.12	−0.007	−0.06	−0.08	−0.03	−0.03	0.10	0.13	−		
AF	0.03	0.04	0.10	0.04	−0.02	−0.04	−0.07	0.33^**^	0.50^**^	0.40^**^	0.17	−	
MVD	0.17	0.18	0.191	0.05	0.08	0.10	0.20	0.52^**^	0.66^**^	0.58^**^	0.48^**^	0.72^**^	−
*M*	2.22	1.76	2.16	20.47	13.25	20.27	12.33	5.22	11.57	14.32	18.65	20.37	1.13
*SD*	0.178	0.185	0.428	3.41	2.59	2.85	4.24	4.60	7.37	8.212	23.37	23.37	0.618

*LOPPBL, Learning outcomes in the preparation of the PBL; LODPBL, Learning outcomes in the defense of PBL; TELO, Test-exam learning outcomes; SMS, Self-knowledge Metacognitive Skills; PMS, Planning Metacognitive Skills; EMS, Evaluation Metacognitive Skills; MS, Motivational Strategies; ACI, Access to Complementary Information; AP, Access to Practices; AT, Access to Theory; ASA, Access to self-evaluation activities; AF, Access to Feedback; MVD, Mean visits per day; M, Mean; SD, Standard Deviation*.

* *p < 0.05*.

** *p < 0.01*.

With regard to the analysis in the CSD, in the first place, we found the existence of relationships between variables (*KMO* = 0.80 and χ^2^ = 523.76, *p* < 0.001). Likewise, significant correlations were found between the results of performance in the different tests and between those and all of the metacognitive skills. Likewise, the pattern of significant correlations coincided with the type of access to the platform and the type of evaluation test. For example, there was a correlation between access to the practices on the platform and the results that the students obtained in the tests of practices (*r* = 0.32, *p* < 0.001). Likewise, the number of visits by day correlates in a significant way with performance in the practices (*r* = 0.35, *p* < 0.01), in the theory (*r* = 0.59, *p* < 0.01) and with the SSM (*r* = 0.39, *p* < 0.01), PMS (*r* = 0.33, *p* < 0.01), EMS (*r* = 0.45, *p* < 0.01), and MS (*r* = 0.32, *p* < 0.01). With regard to participation in the forums, a significant relation was found with the qualification in the theoretical section (*r* = 0.27, *p* < 0.05). Regarding the MS, significant relations were only found with access to feedback actions provided by the teacher (see Table 8).

**Table 8:** **Matrix correlations in CSD, the behaviors on the platform and metacognitive skills and motivational strategies**.

	**1**	**2**	**3**	**4**	**5**	**6**	**7**	**8**	**9**	**10**	**11**	**12**	**13**
LOP	−												
TELO	0.57^**^	−											
SMS	0.62^**^	0.64^**^	−										
PMS	0.53^**^	0.54^**^	0.76^**^	−									
EMS	0.64^**^	0.66^**^	0.90^**^	0.79^**^	−								
MS	0.48^**^	0.48^**^	0.55^**^	0.54^**^	0.57^**^	−							
ACI	0.18	0.31^**^	0.26^*^	0.35^**^	0.33^**^	0.24	−						
AP	0.32^**^	0.40^**^	0.32^**^	0.36^**^	0.37^**^	0.23	0.57^**^	−					
AT	0.21	0.20	0.15	0.20	0.22	0.15	0.45^**^	0.61^**^	−				
ASA	0.09	0.43^**^	0.15	0.29^*^	0.17	0.19	0.46^**^	0.30^*^	0.24^*^	−			
AF	0.39^**^	0.62^**^	0.52^**^	0.57^**^	0.54^**^	0.31^*^	0.41^**^	0.48^**^	0.13	0.52^**^	−		
MVD	0.35^**^	0.59^**^	0.39^**^	0.53^**^	0.45^**^	0.32^**^	0.61^**^	0.60^**^	0.43^**^	0.81^**^	0.73^**^	−	
PF	0.16	0.27^*^	0.11	0.03	0.08	−0.08	−0.02	0.06	0.02	0.26^*^	0.27^*^	0.25	−
*M*	2.11	3.37	15.17	8.87	14.73	8.48	9.84	34.71	17.03	16.41	82.94	14.09	2.11
*SD*	0.88	1.53	5.96	4.09	5.91	5.30	6.97	14.78	12.40	17.49	24.44	14.63	0.88

*LOP, Learning outcomes in the practices; TELO, Test-exam learning outcomes; SMS, Self-knowledge Metacognitive Skills; PMS, Planning Metacognitive Skills; EMS, Evaluation Metacognitive Skills; MS, Motivational Strategies; ACI, Access to Complementary Information; AP, Access to Practices; AT, Access to Theory; ASA, Access to self-evaluation activities; AF, Access to Feedback; MVD, Mean visits per day; PF, Participation in forums; M, Mean; SD, Standard Deviation*.

* *p < 0.05*.

** *p < 0.01*.

### Grouping of students in accordance with the behavioral patterns

The last research question (Will the learning behaviors of the students on the platform offer different learning patterns?) refers to whether the learning behaviors of students on the platform allow us to differentiate between the different types of students. As different patterns of behavior had been noted on the platform, a separate analysis of the clusters in the groups of students studying Health Sciences (OTD and ND) and CSD was performed to corroborate them. In both cases, an Expectation-Maximization algorithm was used (EM) and to determine the appropriate number of clusters, the Bi-Stage Cluster node (hierarchical algorithm based on BIRCH; Zhang et al., 1996) was used with a mean of 0 and a variance of 1.

In the group of students studying health sciences (OTD and ND), only one cluster was identified, which suggests a similar behavior to the other students on the platform.

With regard to the CSD group of students, 3 clusters were detected: Cluster 1 (C1) defined as low (mean between -1.0 and 0; *n* = 36), Cluster 2 (C2) defined as acceptable (mean between 0 and 0.5; *n* = 25) and Cluster 3 (C3) defined as good (mean between 0.5 and 1; *n* = 8) (see Table 9).

**Table 9:** **Centers of final clusters in the CSD**.

	**Cluster**		
	**C1 (Low)**	**C2 (Acceptable)**	**C3 (Good)**
1. Access to complementary information.	7.9	10.8	15.6
2. Access to theory.	16.4	15.0	26.0
3. Access to practice.	31.4	35.7	46.3
4. Access to co-evaluation.	42.7	207.8	542.0
5. Access to feedback provided by the teacher.	72.3	90.3	107.9
6. Participation in the forum.	9.50	13.04	18.63
7. Mean access rate per day.	2.26	3.77	5.73

*C1, Cluster 1; C2, Cluster 2; C3, Cluster 3*.

The second was to determine whether the variables selected as indicators of good use of LMS are equally sustainable in the configuration of the clusters. The three clusters explained a variance of 67.2% [Wilks' Lambda = 0.11; *F*_(14, 120)_ = 17.55, *p* < 0.001, $\eta_{\text{p}}^{2}=0.67$], which implies that the students have different patterns of learning behavior in the three clusters in the seven independent variables. However, not all of the learning behaviors have the same degree of discrimination. In the analysis of the inter-group differentiation, the variables that contributed most to the differentiation were: participation in self-evaluation activities [*F*_(2, 66)_ = 221.18, *p* < 0.000, $\eta_{\text{p}}^{2}=0.87$], mean access rates per day [*F*_(2, 66)_ = 51.85, *p* = 0.000, $\eta_{\text{p}}^{2}=0.61$] and the records of access to feedback provided by the teacher [*F*_(2, 66)_ = 11.350, *p* = 0.000, $\eta_{\text{p}}^{2}=0.26$], and to a lesser degree, records of access to complementary information [*F*_(2, 66)_ = 4.84, *p* = 0.01, $\eta_{\text{p}}^{2}=0.13$], and of access to information on the completion of practices [*F*_(2, 66)_ = 3.64, *p* = 0.03, $\eta_{\text{p}}^{2}=0.10$]. Likewise, neither were significant differences found in records of student access to information on theoretical contents [*F*_(2, 66)_ = 32.57, *p* = 0.08, $\eta_{\text{p}}^{2}=0.07$], nor in participation in forums [*F*_(2, 66)_ = 1.48, *p* = 1.48, *p* = 0.24, $\eta_{\text{p}}^{2}=0.04$].

In addition, the clusters in which the differences were rooted were studied using the Bonferroni difference of means test (see Table 10).

**Table 10:** **Bonferroni Test of Difference of means between the clusters in the variable learning behaviors of CSD students on the platform**.

	**C1 vs. C2**		**C1 vs. C3**		**C2 vs. C3**	
	***DM***	***p***	***DM***	***p***	***DM***	***p***
1. Access to complementary information.	–	–	−7.72	0.01	–	–
2. Access to theory.	–	–	–	–	–	–
3. Access to practice.			−14.81	0.03^*^	–	–
4. Access to co-evaluation.	165.11	0.00^**^	−499.31	0.00^**^	−334.20	0.00^**^
5. Access to feedback provided by the teacher.	−17.97	0.006^**^	35.57	0.00^**^	–	–
6. Participation in the forum.	–	–	–	–	–	–
7. Mean access rate per day.	−1.51	0.00^**^	−3.47	0.00^**^	−1.96	0.00^**^

*C1, Cluster 1; C2, Cluster 2; C3, Cluster 3*.

* *p < 0.05*.

** *p < 0.01*.

With regard to the relation between the patterns of learning on the platform and the learning outcomes (grades), significant differences were found in the results for theoretical aspects [*F*_(2, 69)_ = 5.86, *p* = 0.005, $\eta_{\text{p}}^{2}=0.15$] and in the final grade [*F*_(2, 69)_ = 4.26, *p* = 0.02, $\eta_{\text{p}}^{2}=0.11$], but not in the grades for practical aspects [*F*_(2, 69)_ = 2.89, *p* = 0.06, $\eta_{\text{p}}^{2}$ =0.08]. Likewise, the Bonferroni test was conducted to analyse the clusters between which the differences were found. Differences were found between the cluster defined as good and the clusters defined as low and acceptable and no differences were found between the latter two (see Table 11).

**Table 11:** **Bonferroni test of differences between the means of the Clusters in the learning outcomes for CSD**.

	**C1 vs. C2**		**C1 vs. C3**		**C2 vs. C3**	
	***DM***	***p***	***DM***	***p***	***DM***	***p***
1. Learning outcomes over theoretical aspects.	−	−	−1.92	0.003^**^	−1.57	0.03^*^
2. Learning outcomes over practical aspects.	−	−	−	−	−	−
3. Learning outcomes over global grade.	−	−	−2.32	0.03^*^	−2.49	0.02^*^

*C1, Cluster 1; C2, Cluster 2; C3, Cluster 3*.

* *p < 0.05*.

** *p < 0.01*.

## Discussion and conclusions

The analysis of the learning behaviors of students on the platform is related with the teaching design that the teacher devises. The results confirm differences in the learning behaviors in accordance with the type of B-Learning (Cerezo et al., 2016). These results support the hypothesis that the structuring of teaching influences the learning patterns among students and that there are different patterns in accordance with the type of teaching (RB vs. SB). This information is referential in the interpretation of those patterns of behavior. In the B-Learning environments related with SB, in addition to the information in the behavioral patterns of the students on the platform, it is also necessary to analyse the type and quality of the learning behaviors that the students experience in F2F. So, future studies will address the interactions in these contexts using analytical techniques of protocols for thinking out aloud.

With respect to the relation between the learning behaviors of students on the platform and the learning outcomes, it has been confirmed that there are differences in the learning patterns in the RB group and not so in the SB. These differences also support the hypothesis of the difference in the analysis of the learning behaviors depending on the type of B-Learning (Cerezo et al., 2016). In the RB contexts, the relation has been confirmed between the learning outcomes and the learning behavior on the platform. For example, records of access to practical activities are related to the learning outcomes in the practices and with the results from the evaluation of aspects of theory. Likewise, the completion of self-evaluation activities is related with the results in the evaluation of theory. In summary, the type of evaluation test is related with different behaviors of the student on the platform. This result will help predict the at-risk students and, in addition, will help with the differentiation of the results in the different evaluation tests.

However, no relation was found between the patterns of learning behavior and the learning outcomes in SB. This result indicates that there are variables in F2F environment that would have to be isolated to predict the learning patterns of these students. All of the above implies that not all the variables that have been described as determinants of successful learning on the platform have the same weight. Likewise, not all the learning behaviors are related with success with learning outcomes. Therefore, future investigations will address an analysis of the relation between the learning patterns and the results of students in different evaluation tests. In summary, the procedures for the detection of at-risk students will depend on the B-Learning environment.

Besides, it appears that the pattern of behavior on the platform in the RB model is related with the learning outcomes and with the metacognitive and motivational strategies. In the SB teaching models, a relation has been found between the learning outcomes and the metacognitive responses, but not with the patterns of behavior on the platform, because other F2F learning actions are developed (Cerezo et al., 2016). Likewise, it appears that participation in forums is not a discriminant variable of success at learning, because the teacher takes part in other participative F2F actions in these environments. Therefore, in subsequent investigations, in addition to frequency of participation, its quality will also be analyzed. Also, there are different students' behaviors according to the type of design applied and the degree of virtuality, what could lead to propose different models of platform design depending on the needs of the teacher and the type of teaching (fully virtual, blended or face-to-face). This seems important for the configuration of LMS and for the teacher's approach to the design of the teaching-learning process.

In addition, the relation between learning outcomes, learning behavior on the platform and the metacognitive and the (MS) of students appears to depend on the type of B-Learning (RB vs. SB) and on the type of activity that is proposed to the students. Nevertheless, a relation between the learning outcomes, the planning and EMS and the motivation strategies has been found in the two types of teaching (RB vs. SB). This finding is an important indicator for the teacher in the construction of learning activities on the platform, as the use of these types of strategies can be a predictor of success at learning and can prompt the teacher to conduct training in those areas throughout the instruction process. Future investigations will seek to confirm whether that training produces [e.g., SRL in relation to the execution of the different tasks proposed by the teacher and to the feedback provided through the different evaluation tests designed for learning. For to evaluate self-regulated behaviors, a think aloud protocol (TAP) could be used] improvements in learning outcomes among the students.

With respect to its relation with the behavioral patterns of the students on the platform, there are also differences between RB vs. SB teaching, which supports the results found in the studies of Cerezo et al. (2016) and Zacharis (2015). In RB environments, significant relations have been found between all the metacognitive skills and the learning outcomes, and not in the SB, where no relation has been identified between self-knowledge metacognitive skills and success at learning, which is probably explained by the F2F interaction. With regard to the organization of collaborative forms of teaching, whether in a RB or a SB environment, the use of metacognitive skills has been related with (MS) in students (Bernard and Bachu, 2015; Malmberg et al., 2015; Järvelä et al., 2016; Sáiz and Marticorena, 2016). However, in subsequent studies, an analysis will be conducted to find out whether the type of task that the student has to solve is related to one or another type of metacognitive strategy and what would have to be activated in each case to obtain improvements in learning outcomes.

Likewise, it appears that the learning patterns on the platform discriminate more against at-risk students in RB than in SB. The explained variance in RB teaching was 67.2%. These results are in line with those obtained by Cerezo et al. (2016), Strang (2017), and Zacharis (2015). Nevertheless, not all the variables have the same weight. It appears that the frequency and the systemic approaches of students in their interactions with that platform is a relevant aspect, together with the completion of self-evaluation activities and mean rate of access per day. Hence, as well as frequency, future studies will analyse the type and the quality (actions that the student carries out while accessing the information and how the student processes that information) of the interaction between the learning behaviors of the students on the platform (Yücel and Usluel, 2016).

Another referential aspect is that all of the learning behaviors of the students on the platform are not differentiated by the clusters in a homogenous way. For example, in relation to records of access to complementary information, the distance between the acceptable cluster (C2) and the good cluster (C3) does not differentiate information on theory and feedback provided by the teacher. And in no case, does it differentiate participation in forums. These observations confirm, as we have previously seen, that there is a type of behavior in the learning behavior on the platform that is a function of the type of task that is proposed to the student. This result supports the studies of Park and Il-Hyun (2016) and Harrati et al. (2016) on the differences in behavior on the platform in terms of student characteristics and the structuring of the subject matter by the teachers.

Likewise, the learning behaviors on the platform are not equally well-differentiated, depending on the type of evaluation test that the student is set. This is a referential aspect for future investigations, because it proposes the differentiation of different learning patterns in accordance with the type of evaluation test (Sáiz and Montero, 2016).

In this study, student-teacher, student-content, and student-system interactions have been analyzed. however, in future investigations, student-student and teacher-system relations will be studied, with a view to analyzing whether these behavioral patterns influence the results of student learning (Yücel and Usluel, 2016) and can predict the detection of at-risk students.

All of these conclusions have to be analyzed with prudence in any generalization of the results, as the sample used in this study is not excessively broad and makes reference to students at the same university following three degree courses. Subsequent studies will therefore be directed at enlarging the sample to different populations of university students using the Moodle platform on different degree courses for learning in different B-learning environments. In this study, the variants RB and SB have been analyzed. Likewise, the results in the Flipped blend modality could be included.

## Ethics statement

The ethics committee of the University of Burgos approved this study. Written informed consent was obtained from all participants.

## Author contributions

MM has been the teacher of two of the intervention groups. She also has done theoretical introduction and data analysis. RS, has done the log extraction on the Moodle platform and he performed the theoretical review. Also, he has been the teacher of one of the intervention groups. CO and JP, have supervised the use of data mining techniques on the extracted logs and completed the theoretical review.

## Funding

The work was supported by University of Burgos.

### Conflict of interest statement

The authors declare that the research was conducted in the absence of any commercial or financial relationships that could be construed as a potential conflict of interest.
